# A Lipid-Coated Nanoconstruct Composed of Gold Nanoparticles Noncovalently Coated with Small Interfering RNA: Preparation, Purification and Characterization

**DOI:** 10.3390/nano11112775

**Published:** 2021-10-20

**Authors:** Anna V. Epanchintseva, Julia E. Poletaeva, Ilya S. Dovydenko, Boris P. Chelobanov, Dmitrii V. Pyshnyi, Elena I. Ryabchikova, Inna A. Pyshnaya

**Affiliations:** Institute of Chemical Biology and Fundamental Medicine SB RAS, 630090 Novosibirsk, Russia; annaepanch@niboch.nsc.ru (A.V.E.); fabaceae@yandex.ru (J.E.P.); dovydenko.il@gmail.com (I.S.D.); boris.p.chelobanov@gmail.com (B.P.C.); pyshnyi@niboch.nsc.ru (D.V.P.)

**Keywords:** gold nanoparticles, siRNA, noncovalent adsorption, lipid enveloping, multilayer nanoconstructs for siRNA preparation and purification

## Abstract

There is an urgent need to develop systems for nucleic acid delivery, especially for the creation of effective therapeutics against various diseases. We have previously shown the feasibility of efficient delivery of small interfering RNA by means of gold nanoparticle-based multilayer nanoconstructs (MLNCs) for suppressing reporter protein synthesis. The present work is aimed at improving the quality of preparations of desired MLNCs, and for this purpose, optimal conditions for their multistep fabrication were found. All steps of this process and MLNC purification were verified using dynamic light scattering, transmission electron microscopy, and UV-Vis spectroscopy. Factors influencing the efficiency of nanocomposite assembly, colloidal stability, and purification quality were identified. These data made it possible to optimize the fabrication of target MLNCs bearing small interfering RNA and to substantially improve end product quality via an increase in its homogeneity and a decrease in the amount of incomplete nanoconstructs. We believe that the proposed approaches and methods will be useful for researchers working with lipid nanoconstructs.

## 1. Introduction

Nucleic-acid therapeutics have tremendous therapeutic potential for the treatment of many diseases. Nonetheless, the number of such therapeutics approved for clinical use is still low [1]. First of all, the reason is the nature of nucleic acids, which are macromolecules with a high negative charge and therefore are unable to penetrate into the cell on their own [2]. In addition, nucleic acids are very sensitive to nucleases, as evidenced by the low stability of nucleic acids in physiological fluids [3]. These obstacles can be overcome by (i) the development of modifications of nucleic acids and (ii) the creation of delivery systems that ensure not only penetration into the cell but also longer half-life of the nucleic acid formulation in the human body.

During the past several decades, a variety of systems of nucleic acid delivery have been proposed [4,5,6,7,8]. A distinct field of research and development of these systems is the use of solid (metal) nanoparticles enclosed in a shell and serving as a carrier of a nucleic acid. For instance, there are reports on the efficient delivery of small interfering RNA (siRNA) via gold nanoparticles (AuNPs) [9] and selenium nanoparticles [10] coated with chitosan and gold nanorods covered with two layers of polyelectrolytes [11].

**Figure 1:**
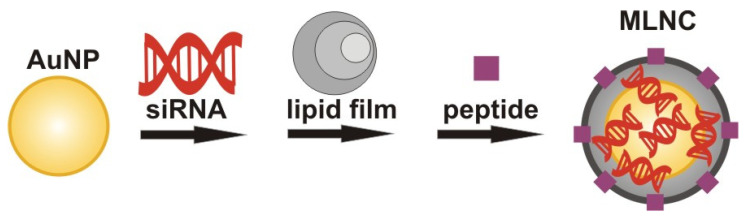
The fabrication and structure of the desired MLNC.

Our study was aimed at creating multilayer nanoconstructs (MLNCs) based on spherical AuNPs on whose surface an siRNA layer is adsorbed and surrounded by a lipid envelope (Figure 1). The choice of AuNPs as the basis for this nanocomposite is due to their unique physicochemical properties and good biocompatibility [12,13,14].

In the present study, the fabrication of an MLNC includes several steps: preparation of core nanoparticles (AuNP-siRNA) and a lipid film, assembly of the MLNC, and purification and concentration of the nanoconstruct particles. One of the previously developed versions of an MLNC—Whose lipid envelope is doped with peptide (LR)_4_G conjugated with stearic acid—Successfully delivers siRNA into cells and ensures a release of the siRNA cargo, as confirmed by the suppression of reporter protein expression (green fluorescent protein; GFP) [15]. In that study, we showed the feasibility of siRNA delivery via constructs containing lipid-coated AuNPs [15]. On the other hand, designing formulations intended for clinical use requires higher nanoconstruct quality, which can be achieved by improving the method of MLNC fabrication and will enhance the efficiency of siRNA delivery into human cells.

The second aim of this work was to optimize the conditions of MLNC synthesis that yield the most homogeneous suspensions free of empty lipid particles, naked core nanoparticles, and aggregates of core nanoparticles. To increase the efficiency of MLNC synthesis, we varied the conditions of all steps of this process on the basis of our previously obtained and published data about the stability and properties of nanoconstructs. In particular, we varied the ratio of core nanoparticles to a lipid mixture; an optimal buffer was chosen that enables effective wrapping of the core nanoparticles with a lipid envelope; and optimal conditions were found for the fractionation and purification of the MLNCs.

## 2. Materials and Methods

### 2.1. Chemicals

Tetrachloroauric acid trihydrate (HAuCl_4_·3H_2_O) was purchased from Aurat (Moscow, Russia), and RNA phosphoramidites for oligoribonucleotide synthesis were acquired from Sigma-Aldrich (Hamburg, Germany). Sodium chloride (NaCl) and magnesium sulfate (MgSO_4_) were bought from Honeywell (Seelze, Germany) and sodium citrate dihydrate (Na_3_C_6_H_5_O_7_·2H_2_O) from Fluka (Buchs, Switzerland). Magnesium acetate tetrahydrate [Mg(CH_3_COO)_2_·4H_2_O] was acquired from VWR International LLC (Radnor, PA, USA), whereas egg phosphatidylcholine and 1,2-dioleoyl-sn-glycero-3-phosphoethanolamine (DOPE) was obtained from Avanti (Alabaster, AL, USA). 2-[[4-Dodecylamino-6-oleylamino-1,3,5-triazine-2yl]-(2-hydroxyethyl)amino]ethanol (DOME2) was synthetized as described in ref. [13]. Disodium phosphate dihydrate (NaH_2_PO_4_·2H_2_O) and monosodium phosphate dodecahydrate (Na_2_HPO_4_·12H_2_O) were purchased from Reatex (Moscow, Russia) and uranyl acetate from SPI (West Chester, PA, USA). Peptide NH_2_-(RL)_4_G-C(O)NH_2_·5CF_3_COOH was acquired from Diapharm (Lyubertsy, Russia). Sodium acetate trihydrate (CH_3_COONa·3H_2_O), acetic acid (CH_3_COOH), and sucrose (C_12_H_22_O_11_) were bought from Panreac (Barcelona, Spain), whereas cesium chloride (CsCl) and glycerol (C_3_H_8_O_3_) were obtained from Applichem (Darmstadt, Germany), and trichloromethane (chloroform, CHCl_3_) and methanol (CH_3_OH) were purchased from Reachem (Moscow, Russia). AlamarBlue™ Cell Viability Reagent was acquired from Invitrogen (Waltham, MA, USA). Water was purified by means of a Simplicity 185 water purification system (Millipore, Burlington, MA, USA) and had a resistivity of 18.2 MΩ·cm at 25 °C.

### 2.2. Preparation of Core Nanoparticles

To obtain core nanoparticles (AuNP-siRNA), siRNA was used that suppresses GFP synthesis in cultured cells stably expressing this protein. The sequence of the siRNA was as follows: sense strand, 5′-CAAGCUGACCCUGAAGUUCTT; and antisense strand, 5′-GAACUUCAGGGUCAGCUUGTT [16]. The synthesis of this siRNA is described in detail in our previous work [17]. The AuNPs were synthesized using a previously published technique [18].

The core nanoparticles, which are AuNPs noncovalently covered with siRNA, were fabricated as described elsewhere [15,19], with some modifications: 4 mM citrate–stabilized AuNPs (size 12 ± 1 nm according to transmission electron microscopy [TEM] or 17.3 ± 2.1 nm according to dynamic light scattering [DLS] analysis; a zeta potential of −33.6 ± 2.0 mV according to DLS) at a concentration of 3.6 × 10^−9^ M were incubated with 0.72 μM siRNA for 22 h at room temperature in the presence of either 5 or 10 mM NaCl and either Mg(CH_3_COO)_2_ or MgSO_4_. The resultant suspensions of core nanoparticles were centrifuged, the supernatant was discarded, and the pellet containing the core nanoparticles was washed with 1 mL of 4 mM sodium citrate dihydrate. The size and monodispersity of the obtained core nanoparticles were determined using TEM (13 ± 1 nm) and DLS (25 ± 9 nm). The zeta potential of the core nanoparticles proved to be −44 ± 1 mV, and the polydispersity index (PDI) of the suspension was 0.212 ± 0.011. The core nanoparticles had a surface plasmon resonance maximum at 520 nm, and the density of siRNA molecules on the AuNP surface was 29 ± 6.

### 2.3. Preparation of a Lipid Film

The lipid film was prepared as described before [15], with modifications: 90 μL of 1 mM egg phosphatidylcholine and DOPE in a CHCl_3_/CH_3_OH mixture (1:1) and 10 μL of 1 mM DOME2 in CHCl_3_ were added to 1 mL of CHCl_3_ in a 10 mL round-bottom flask. Next, the solvent was evaporated either at 25 °C and 6 mmHg or without thermostatting at 12 mmHg. The resultant lipid film was then dried in vacuum in a desiccator to remove traces of the organic solvents, after which the flask with the film was incubated at −18 °C for 16 h. All the procedures for obtaining the lipid film were carried out in an argon atmosphere.

### 2.4. Synthesis of a Peptide Conjugate with Stearic Acid

A conjugate of a peptide [(RL)_4_G-NH_2_] with stearic acid [Str-(RL)_4_G-NH_2_] for doping the lipid envelope was synthesized as described previously [15].

### 2.5. Assembly of the MLNC

The procedure was performed as described earlier [15], with slightly modified reaction conditions, as described below. The MLNCs were obtained in two stages:

First, a buffer with pH 4.5 was applied to the surface of the lipid film: either (1) 0.9 mL of H_2_O and 31 μL of NaH_2_PO_4_ (1.00 M, 0.10 M, or 0.01 M) or (2) 0.825 mL of H_2_O; to the buffer, we added 100 μL of a suspension of the core nanoparticles (2.5 pmol in terms of gold) in 6 mM CH_3_COOH. The reaction mixture of the core nanoparticles and lipid film was sonicated at 90 W for 15 min at 25 °C. The expected result of this step is a suspension of the core nanoparticles bearing a layer of noncovalently attached siRNA enclosed in a lipid envelope.

Second stage: the pH of the suspension containing the core nanoparticles carrying the lipid envelope was adjusted to 7.4 by the addition of either (1) 69 μL of 1.00, 0.10, or 0.01 M Na_2_HPO_4_ or (2) 100 μL of 3 mM CH_3_COONa. Then, 10 μL of 1 mM stearic-acid-conjugated peptide, Str-(LR)_4_G, was introduced. The reaction mixture was sonicated (90 W) for 5 min at 25 °C. The size and monodispersity of the obtained MLNCs were verified using TEM and DLS. The MLNCs had a surface plasmon resonance maximum at 534 nm.

Thus, we obtained a suspension of MLNCs whose envelope was doped with a peptide. For brevity, hereafter, these nanoparticles are referred to as “MLNCs,” without a mention of the peptide.

### 2.6. Purification of the MLNCs by Banding Centrifugation

Fractionation of the MLNCs was carried out either {1} in a cesium chloride gradient or {2} in solutions of glycerol or sucrose.

{1} In a 1.5 mL test tube, we sequentially layered 500 μL each of an aqueous 61.7% CsCl solution (ρ = 1.789 g/cm^3^, μ = 1.238 9 cP) and an aqueous 10.8% CsCl solution (ρ = 1.0804 g/cm^3^, μ = 0.966 cP) and then 50 μL of the MLNC suspension; its colloidal stability was examined by means of changes in the color of the MLNC layer.

{2} In a 15 mL test tube, we layered 500–1000 μL of the MLNC suspension on top of 10 mL of (i) an aqueous 75% glycerol solution (μ = 41 cP) or (ii) an aqueous 26% sucrose solution in 1 mM phosphate buffer (pH was altered) (ρ = 1.1082 g/mL, μ = 2.223 cP) or (iii) an aqueous 58% sucrose solution in 1 mM phosphate buffer (ρ = 1.267 g/mL, μ = 42.8 cP), followed by centrifugation at 25 °C in the range of 1000–8000× *g* for 15–90 min. Fractions of different colors were collected into separate 1.5 mL tubes and were subjected to purification to remove excess glycerol or sucrose via dialysis or centrifugation. After that, all the fractions were analyzed using DLS and TEM.

After the fractionation in sucrose, the samples with or without an added equal volume of 1 mM phosphate buffer were centrifuged in 1.5 mL tubes at 2000× *g* for 15, 30, or 60 min, the supernatant was discarded, and the resulting MLNCs were analyzed via DLS, TEM, and UV-Vis spectroscopy.

After the fractionation in glycerol, dialysis was performed either in Centricon Plus-70 3 kDa, Merck KGaA (Darmstadt, Germany) or in screw cap 1.5 mL tubes at 25 °C for 16–72 h against 1, 3, or 10 mM phosphate buffer using either (1) membranes for extruders with a pore diameter of 30 or 100 nm, Whatman (Maidstone, UK) or (2) single-layer 3.5 or 10 kDa dialysis bags, Thermo Fisher Scientific (Göteborg, Sweden).

### 2.7. Examination of the Composition and Quality of the Samples

#### 2.7.1. Optical Extinction Spectra

**Figure 2:**
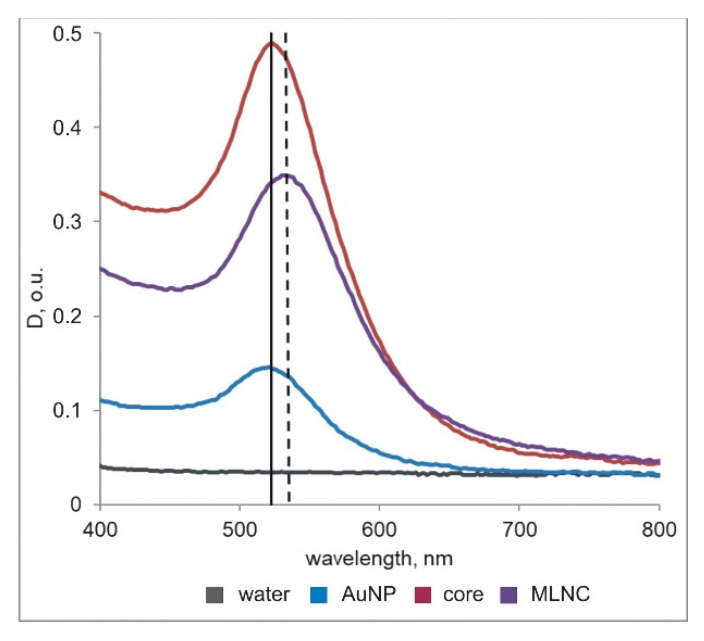
“Typical” spectra of the obtained samples of AuNPs, core nanoparticles, and MLNCs. The vertical lines indicate absorption maxima for AuNPs and core nanoparticles (520 nm, left-hand line) and for MLNCs (532 nm, right-hand line). For clarity, we present the spectra of the samples at concentrations that ensure resolution of the lines in the graph.

To verify colloidal stability and to determine gold concentration, we performed UV-Vis spectroscopy. Optical adsorption spectra of the core nanoparticles and all preparations of MLNCs (Figure 2) were recorded on a Clariostar plate fluorimeter, BMG Labtech (Ortenberg, Germany) in the range 400–800 nm according to the manufacturer’s instructions. The absorption maximum of all versions of MLNC preparations was found to be shifted by 12 nm relative to AuNPs and core nanoparticles; this shift emerged after the lipid envelope formation and was not affected by other experimental conditions. Preservation of colloidal stability of the samples during the procedure was confirmed by the absence of a peak in the range 600–700 nm.

The yield of MLNCs was calculated from optical density of the final suspension at 520 nm using the molar extinction coefficient characteristic of the original AuNPs (ε = 8.78 × 10^8^ L·mol/cm) [20]. The amount (mol) of AuNPs used to fabricate the core nanoparticles was set to 100%.

#### 2.7.2. DLS

All samples of core nanoparticles and MLNCs were characterized via DLS and TEM. DLS analysis is employed for assessing overall hydrodynamic characteristics of a suspension of nanoparticles, whereas TEM provides information on the fine structure of nanoparticles and its changes. These methods complement each other and, taken together, help to exhaustively characterize samples of nanoconstructs.

Suspensions of the core nanoparticles and MLNCs were analyzed using DLS by means of a Malvern Zetasizer Nano instrument, Malvern Instruments (Worcestershire, UK) in accordance with the manufacturer’s instructions. This method allowed us to characterize particle suspensions in terms of the following parameters: the hydrodynamic diameter, PDI, and zeta (ζ) potential. The measurements were performed at least in triplicate.

#### 2.7.3. TEM

For examination using TEM, 10 μL of a suspension of the core nanoparticles or MLNCs was applied to a formvar film on a grid and incubated for 1 min. Then, the drop was aspirated with a pipettor, and without drying, the grid was placed on a drop of uranyl acetate for 10 s; the excess liquid was removed with filter paper. All TEM samples were prepared identically; at least five grid cells were examined in different parts of each grid. The suspensions of the core nanoparticles and MLNCs were investigated under a JEM 1400 transmission electron microscope, Jeol (Tokyo, Japan) equipped with a Veleta digital camera, EM SIS (Muenster Germany). Particle sizes were determined using iTEM software, version 5.2, EM SIS (Muenster, Germany).

### 2.8. Cytotoxicity Assays of Glycerol and Sucrose

To this end, SC-1 R780 fibroblasts were cultured in the IMDM medium, Gibco (Grand Island, NY, USA) supplemented with 10% of fetal calf serum (Gibco), penicillin (100 U/mL), and streptomycin (100 μg/mL) in a humidified atmosphere containing 5% of CO_2_ at 37 °C. The cytotoxicity assay was performed using the AlamarBlue test [21].

### 2.9. Statistical Analysis

Each experiment was conducted independently at least three times. Data are presented as mean ± standard deviation from at least three independent experiments.

## 3. Results

### 3.1. The Mechanism of MLNC Formation

The formation of a lipid envelope around the core nanoparticle is mediated by electrostatic interaction between protonated amino groups of the lipid (DOME2, which is a component of the lipid film) and negatively charged phosphate groups of the siRNA on the surface of the core nanoparticle. Because DOME2 is protonated at a pH below 7, the assembly of the nanoconstructs was carried out in an acidic medium. This strategy necessitated selecting a buffer that makes it easy to switch DOME2 to either a charged (protonated) state or a neutral (deprotonated) state and ensures colloidal stability of the core nanoparticles. We believe that choosing such a buffer will reduce the aggregation of MLNC particles and aggregation of “naked” core nanoparticles.

**Figure 3:**
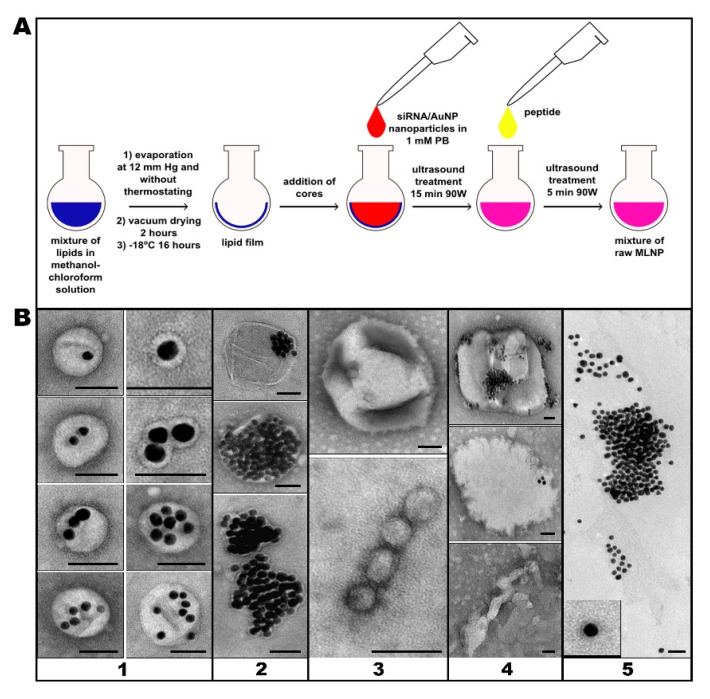
(**A**) The scheme of MLNC formation after the application of a suspension of the core nanoparticles to the lipid film and sonication. (**B**) Morphology of the particles in the MLNC preparations. Panel 1: Different subtypes of a “target MLNC” containing 1 to 10 electron-dense core nanoparticles; Panel 2: Aggregates of the core nanoparticles surrounded by a thin lipid envelope; Panel 3: Empty lipid particles; Panel 4: Fragments of the lipid film with adhered core nanoparticles; Panel 5: “Naked” core nanoparticles. The scale bars correspond to 50 nm. Negative staining with uranyl acetate followed by TEM.

To form a lipid envelope around the core nanoparticle, a suspension of the core nanoparticles in an acidic buffer was applied to the prepared lipid film, and the reaction mixture was sonicated (Figure 3A). The lipid film, which carries the core nanoparticles adsorbed on the surface, gets fragmented, and the core nanoparticles end up in a lipid envelope. The sizes of the forming particles are different, as are the numbers of core nanoparticles enclosed in a single shell. Consequently, the resulting suspension contains structurally diverse desired MLNCs (“target MLNCs”: Figure 3B, panel 1), aggregates of the core nanoparticles surrounded by a thin lipid envelope (Figure 3B, panel 2), lipid particles and empty vesicles (Figure 3B, panel 3), lipid film fragments of various sizes with adhered core nanoparticles (Figure 3B, panel 4), and naked core nanoparticles, both stand-alone and aggregated (Figure 3B, panel 5).

It is obvious that the ideal end product should consist mainly of stand-alone MLNCs each containing one core nanoparticle. The presented mechanism of MLNC formation makes this outcome a pipe dream, mainly owing to the heterogeneity of adsorption of the core nanoparticles on the lipid film. Therefore, our efforts were focused on obtaining suspensions with the highest proportion of stand-alone MLNCs containing 1–10 core nanoparticles and a distinct electron-transparent envelope; such nanoconstructs are hereinafter referred to as “target MLNCs” (Figure 3B, panel 1). Meanwhile, we strived to reduce the concentrations of empty lipid particles, aggregates of core nanoparticles enclosed in a thin lipid envelope, and naked (unenveloped) core nanoparticles (Figure 3B, panels 2–5). It should be noted that we always assessed the effect of one or another modification of the protocol by means of changes in the characteristics of the end product: the final suspension of MLNCs.

### 3.2. Assembly of the Core Nanoparticles: AuNP-siRNA

The first step in the fabrication of MLNCs is the synthesis of core nanoparticles (AuNP-siRNA); we have described this procedure in detail previously [15,22,23]. For enveloping the core nanoparticles with the siRNA, a suitable buffer was experimentally selected, phosphate buffer, which enabled easy switching from pH 5 to pH 7.4, which corresponds to physiological pH. It is important that this buffer ensured colloidal stability of the core nanoparticles throughout the entire experiment on MLNC fabrication.

**Figure 4:**
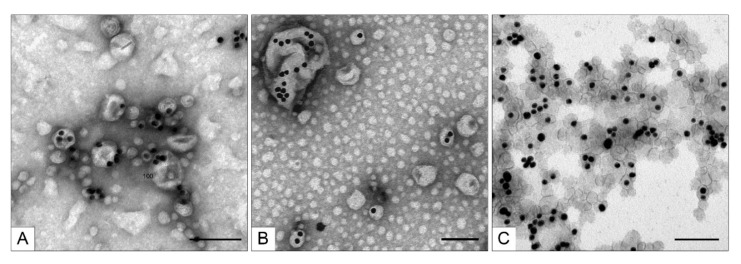
Ultrastructure of the obtained MLNCs. (**A**) Assembly in 100 mM phosphate buffer, (**B**) assembly in 10 mM phosphate buffer, and (**C**) assembly in 1 mM phosphate buffer. The scale bars correspond to 100 nm. Negative staining with a 0.5% uranyl acetate solution followed by TEM.

During the preparation of MLNCs, it became apparent that the lipid particles, which contained lipid DOME2, had negative ζ potential in acidic phosphate buffer, indicating adsorption of phosphate anions on the surface of the lipid film. Accordingly, the phosphate component of the buffer will compete with the core nanoparticles during their adsorption onto the lipid film, thereby inevitably decreasing the efficiency of the enveloping process. At first glance, the simplest solution seems to be to change the buffer, but this approach can diminish colloidal stability of the core nanoparticles. We reduced the phosphate anion concentration from 0.100 to 0.001 M, which increased the efficiency of MLNC formation (Figure 4) and decreased the PDI (Table 1).

By lowering the buffer concentration, we reduced the amount of phosphate anions adsorbed on the lipid film surface; however, it was necessary to neutralize the effect of the remaining ones. For this purpose, it was decided to add a divalent cation that would act as a linker between the phosphate anions adsorbed on the lipid film surface and the siRNA on the surface of AuNPs. Mg^2+^ was chosen and did not lead to “adhesion” and precipitation of the components of the lipid film and core nanoparticles, nor does it catalyze phosphodiester bond hydrolysis in siRNA. Nevertheless, which salt can be used in this context? It is known that the type of anion has a pronounced effect on colloidal stability of core nanoparticles [24,25,26]. Accordingly, we introduced either magnesium acetate or magnesium sulfate into the reaction mixture.

During the synthesis of the core nanoparticles, the influence of Mg(CH_3_COO)_2_ or MgSO_4_ at 0.1 or 0.4 mM added to the reaction mixture was determined by means of physicochemical characteristics (Table 2) and alterations of colloidal stability of the core nanoparticle suspension. Acetate ions at both concentrations affected the colloidal stability negatively. The addition of magnesium ions in the form of 0.1 mM sulfate “improved” hydrodynamic parameters of the core nanoparticles and did not alter colloidal stability for several days. At 0.4 mM, the impact of this salt was negative.

**Figure 5:**
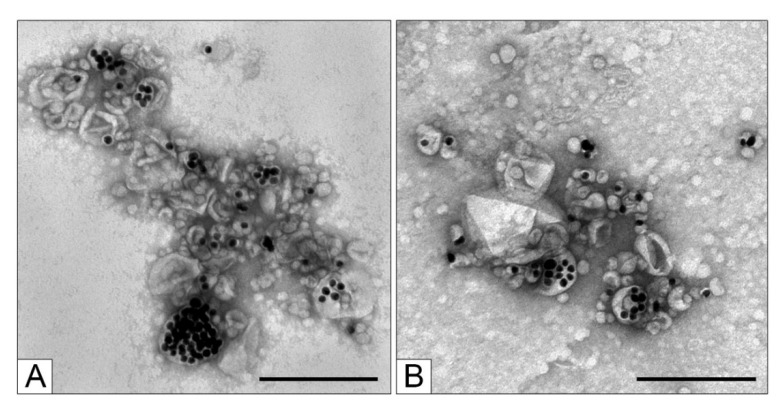
Ultrastructure of the MLNCs obtained in the presence of 0.1 mM MgSO_4_. (**A**) MLNCs prepared via the addition of 10 mM NaCl or (**B**) 5 mM NaCl. The scale bars correspond to 200 nm. Negative staining with a 0.5% uranyl acetate solution followed by TEM.

Having found the optimal concentration of MgSO_4_ (0.1 mM) for obtaining the core nanoparticles, we began to vary the concentration of NaCl, the presence of which is necessary for denser loading of siRNA molecules onto AuNPs. AuNPs are very sensitive to monovalent anions [25], and even a small shift in their concentration can affect colloidal stability of the resulting core nanoparticles. Previously [15], we prepared core nanoparticles at 10 mM NaCl (parameters of the obtained preparations of MLNCs: hydrodynamic diameter of 205 ± 100 nm and PDI of 0.186), but in the present work, we reduced the NaCl concentration in half, which increased suspension homogeneity and reduced the size of MLNCs (hydrodynamic diameter became 128 ± 54 nm, and PDI became 0.151). The change in NaCl concentration did not lead to a noticeable alteration of the end products’ composition according to TEM; they contained MLNCs with different numbers of core nanoparticles as well as lipid particles, empty lipid vesicles, aggregates of the core nanoparticles, and a small percentage of naked core nanoparticles (Figure 5). In subsequent experiments, the reaction mixture version containing 0.1 mM MgSO_4_ and 5 mM NaCl was utilized to synthesize the core nanoparticles.

### 3.3. Fabrication of the Lipid Film

**Figure 6:**
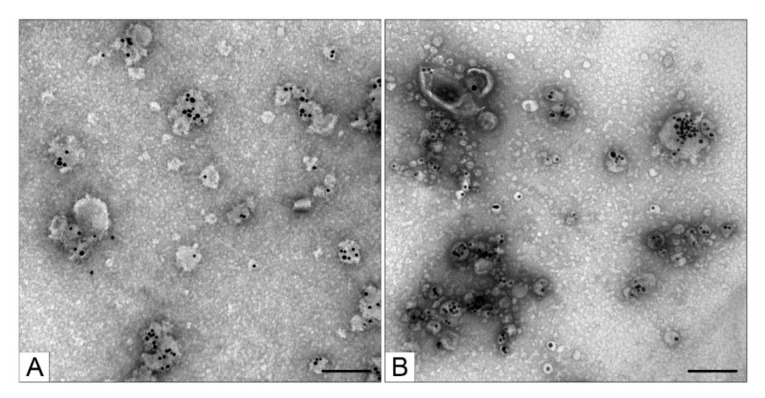
Ultrastructure of the MLNCs prepared from the lipid film synthesized under a pressure of 12 mmHg (**A**) or 6 mmHg (**B**). The scale bars correspond to 200 nm. Negative staining with a 0.5% uranyl acetate solution followed by TEM.

The lipid film was generated via evaporation of a solution of mixed lipids in a chloroform/methanol mixture under reduced pressure (6 mmHg). We found that the evaporation rate, which can be adjusted by changing pressure, affects the quality of the resulting film: an increase in pressure from 6 to 12 mmHg and elimination of flask thermostatting improved the homogeneity of the lipid film and facilitated its interaction with the core nanoparticles. Accordingly, more homogeneous end products with a higher concentration of MLNCs were obtained. For instance, the hydrodynamic diameter of MLNCs decreased from 188 ± 96 to 144.3 ± 79 nm, while the PDI was 0.259 ± 0.005 nm and 0.250 ± 0.006 nm, respectively. TEM analysis of these samples revealed no appreciable differences in their composition: they contained MLNCs of various structures, empty lipid particles, aggregates of the core nanoparticles within a thin lipid envelope, and naked core nanoparticles (Figure 6).

### 3.4. Doping of the Lipid Envelope of MLNCs with the Peptide

The final step in the fabrication of the target MLNCs is doping of the lipid envelope with peptide Str-(LR)_4_G at pH 7.4 to make sure that the nanoparticles can penetrate into the cell and to overcome endosomal arrest as well as to enable the release of the siRNA into the cytosol from the surface of the core nanoparticle. The effectiveness of this procedure has been demonstrated by us previously [15]. In the present work, a sample of lipid-coated core nanoparticles obtained at pH 4.5 via sonication was transferred to a medium with pH 7.5, after which the peptide was added, and the mixture was sonicated for 5 min at 25 °C.

It turned out that the doping of the lipid envelope with Str-(LR)_4_G affects physicochemical parameters of the resultant MLNCs: the addition of the peptide yielded an increase in the hydrodynamic diameter from 118.4 ± 44.27 to 235.1 ± 100.3 nm, while the PDI was 0.1942 and 0.1854 for the MLNCs without doping and after the doping, respectively. Structural analysis of the end product using TEM did not uncover any noticeable changes in its composition and in particle structure of the MLNCs after the doping with peptide Str-(LR)_4_G.

**Figure 7:**
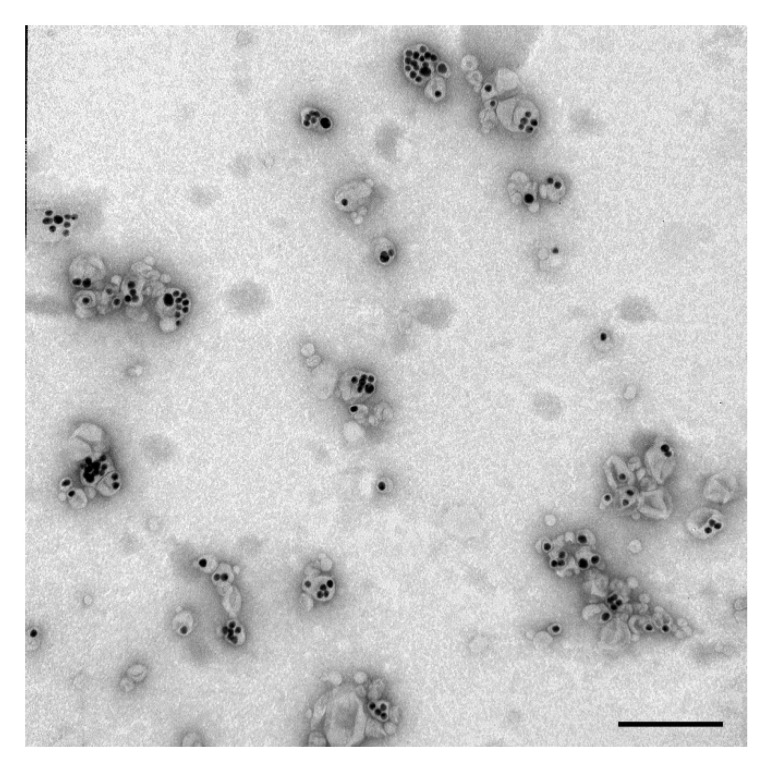
Ultrastructure of optimized MLNCs. The scale bar corresponds to 200 nm. Negative staining with a 0.5% uranyl acetate solution followed by TEM.

These findings allowed us to find optimal modifications of the initial protocol [15] that afford the best quality of MLNCs: the presence of 0.1 mM MgSO_4_ and 5 mM NaCl during the assembly of the core nanoparticles, lipid film synthesis at 12 mmHg without thermostatting, and the assembly of MLNCs in 1 mM phosphate buffer. The end product obtained under these conditions is hereinafter referred to as “optimized MLNCs” (Figure 7). The preparations of optimized MLNCs are characterized by a hydrodynamic diameter of 152 ± 75 nm and a PDI of 0.201 ± 0.012.

The presented modifications of the protocol of MLNC fabrication increased the efficiency of synthesis of core nanoparticles carrying the lipid envelope, but the obtained suspensions still contained a noticeable amount of lipid particles and vesicles, aggregates of the core nanoparticles within a thin lipid envelope, and naked core nanoparticles, thus necessitating purification of the MLNC preparations. Given that the MLNCs, lipid particles, and core nanoparticles have different densities and sizes, they can be separated using banding high-speed centrifugation.

### 3.5. Fractionation of MLNCs via Centrifugation

Cesium chloride density gradient fractionation is the best-studied and widely used technique for the separation of macromolecules. We fractionated optimized MLNCs in a stepwise CsCl gradient; as a consequence, the color of the sample turned from dark red to blue, indicating complete loss of colloidal stability of these nanoparticles in the cesium chloride solution.

After giving up on cesium chloride, for the fractionation of MLNCs, we chose an aqueous solution of sucrose as a viscous and less “aggressive” medium. The fractionation was conducted in a homogeneous solution without a concentration gradient. The sucrose concentration was selected according to calculations suggesting that target MLNCs should penetrate into the viscous medium to 2 cm depth. The computation was performed using a calculator [27]. To this end, the density of the nanoparticles was assumed to equal that of gold, 19 g/cm^3^, and we hypothesized that during the centrifugation, the less dense envelope and the heavy dense core would move at different acceleration rates. In accordance with the calculation results, a 58% sucrose solution was selected (ρ = 1.267 g/mL, µ = 42.8 cP).

On top of the sucrose solution, 500–1000 μL of the optimized MLNC suspension was layered, followed by centrifugation at 25 °C and 2000× *g* for 1 h. The resultant fractions were carefully collected with a dispenser. The high concentration of sucrose makes the collected fractions unsuitable for examination using TEM and accordingly for assessing their quality. To remove the excess sucrose, an equal volume of 1 mM phosphate buffer was added to the samples of selected fractions of MLNCs; the resulting suspensions were centrifuged for 15 min at 2000× *g*. The supernatant was discarded, the pellet was resuspended in 1 mM phosphate buffer, and this sample was analyzed via TEM and DLS.

**Figure 8:**
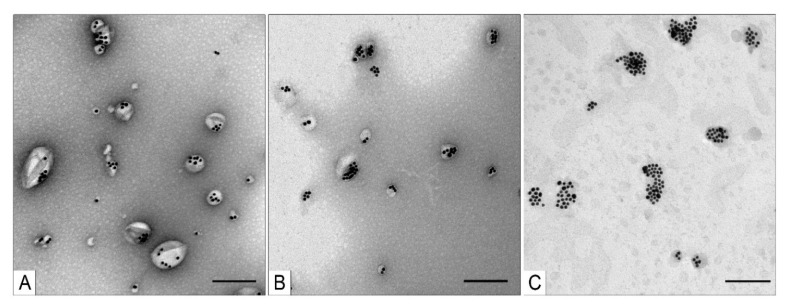
Ultrastructure of the fractions of optimized MLNCs obtained via centrifugation in a 58% sucrose solution for 1 h at 25 °C and 2000× *g*. (**A**) Upper fraction, (**B**) middle fraction, and (**C**) bottom fraction. The scale bars correspond to 200 nm. Negative staining with a 0.5% uranyl acetate solution followed by TEM.

The TEM analysis of the fractions obtained via centrifugation in the 58% sucrose solution showed that the middle and upper fractions were very similar because target MLNCs constituted the bulk of each sample (Figure 8A,B). By contrast, the bottom fraction mainly consisted of large aggregates of the core nanoparticles enclosed in a thin lipid envelope and aggregates of naked core nanoparticles (Figure 8C).

We evaluated the yield of target MLNCs after all the steps of fabrication and purification. For this purpose, the gold content of the target fraction was calculated from its optical density and then divided by the amount of gold in the AuNPs used for the synthesis of the core nanoparticles. The yield of MLNCs (in terms of gold) in the middle fraction (Figure 8B) was 15%; the sample after the removal of excess sucrose contained 5.5% of sucrose, and the hydrodynamic diameter of particles in this fraction was 195 ± 78 nm with a PDI of 0.173.

The low yield of MLNCs may be related both to the low efficiency of coating of the core nanoparticles during the fabrication of MLNCs and to degradation of the MLNC sample during the fractionation because the heavy gold core can “pierce” the lipid envelope during the centrifugation. Consequently, centrifugation duration was reduced to 30 min, which yielded four fractions represented by colored discrete rings. Characteristics of these fractions are given in Table 3. TEM analysis of the fractions did not reveal any appreciable differences in the composition and structure of their nanoparticles as compared with the MLNC preparations after 1 h centrifugation.

The total gold content of the four fractions was 68% of the initial gold amount in AuNPs (utilized for the synthesis of core nanoparticles). One-third of the gold (apparently in the form of naked core nanoparticles released during the disintegration of MLNCs) was distributed throughout the rest (10 mL) of the sucrose solution volume in the centrifuge tube and was not detectable by direct observation.

**Figure 9:**
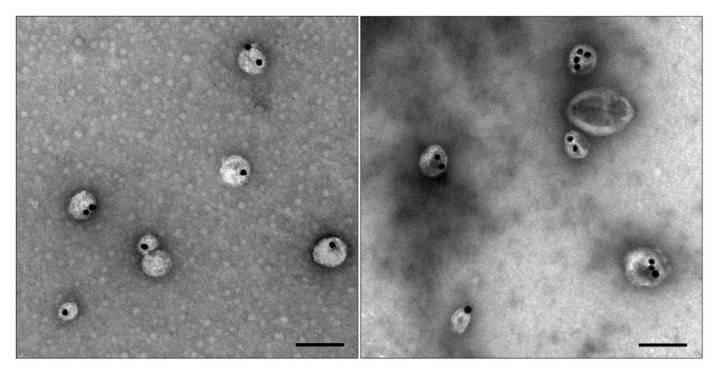
Ultrastructure of optimized MLNCs after 15 min fractionation in 58% sucrose and removal of excess sucrose. The scale bars correspond to 100 nm. Negative staining with a 0.5% uranyl acetate solution followed by TEM.

In search of optimal fractionation duration, we reduced the centrifugation duration to 15 min and noticed that this time enables MLNCs’ separation into two discrete fractions. The upper fraction contained 13–15% of MLNCs, which corresponds to the upper fraction with the half-hour centrifugation. At the same time, an increase in the concentration of MLNCs up to 40% was observed in the middle fraction. It is this fraction (Figure 9) that we are currently testing in cell culture assays.

Our study indicates that the amount of target MLNCs in the final suspension is determined not only by the efficiency of coating of the core nanoparticles with the lipid envelope but also by MLNC preservation during the purification and concentration procedures. We believe that the approaches that we used to improve the quality of lipid-coated nanoconstructs and the newly developed methodology will be useful to researchers creating similar nanoconstructs.

Undoubtedly, an important factor in the proposed method is the choice of a medium for separating a suspension of MLNCs into fractions. In addition to sucrose and cesium chloride, we tested another type of viscous medium: aqueous solutions of glycerin (details are given in the Materials and Methods section).

**Figure 10:**
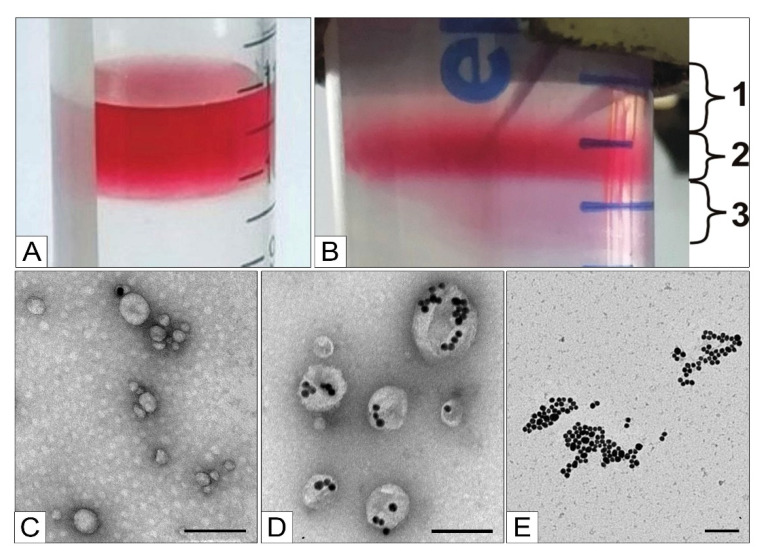
Top row: purification of optimized MLNCs via centrifugation in 75% glycerin. (**A**) The sample is applied to a glycerin layer; (**B**) separation of the sample into three fractions after centrifugation (1: upper fraction, 2: middle (target) fraction, and 3: bottom fraction). Bottom row: fractions of optimized MLNCs obtained via centrifugation in 75% glycerol and purified via dialysis: (**C**) upper fraction, (**D**) middle fraction, and (**E**) bottom fraction. The scale bars correspond to 100 nm. Contrasting by means of a 0.5% uranyl acetate solution followed by TEM.

The best results were obtained with the following parameters of the procedure: centrifugation in a 75% glycerol solution for 40 min at 8000× *g* and 25 °C with subsequent dialysis for 16 h (using membranes for extruders, pore diameter 30 nm). As in the case of sucrose, in glycerol solutions, optimized MLNCs get separated into fractions (Figure 10A). According to TEM (Figure 10B), the upper fraction contained lipid particles and vesicles (hydrodynamic diameter 123 ± 55 nm, PDI = 0.366), the middle dark-red fraction contained MLNCs (hydrodynamic diameter 127 ± 40 nm, PDI = 0.279), and the bottom fraction consisted of large aggregates of the core nanoparticles enclosed in an “incomplete” lipid envelope and aggregates of core nanoparticles stuck to fragments of lipid envelopes (hydrodynamic diameter 233 ± 74 nm, PDI = 0.280).

We fine-tuned all stages of the fractionation of optimized MLNCs in glycerol solutions, subsequent purification via dialysis, and the concentration procedure (data not shown). The highest-quality end products were homogeneous, had a PDI of ~0.160, and contained ≥22–27% of glycerol as well as nanoparticles with a hydrodynamic diameter of 115 ± 49 nm. There were concerns that the presence of glycerol could negatively affect cell viability, and we performed an assay of its cytotoxicity on cultured SC-1 R780 fibroblasts.

**Figure 11:**
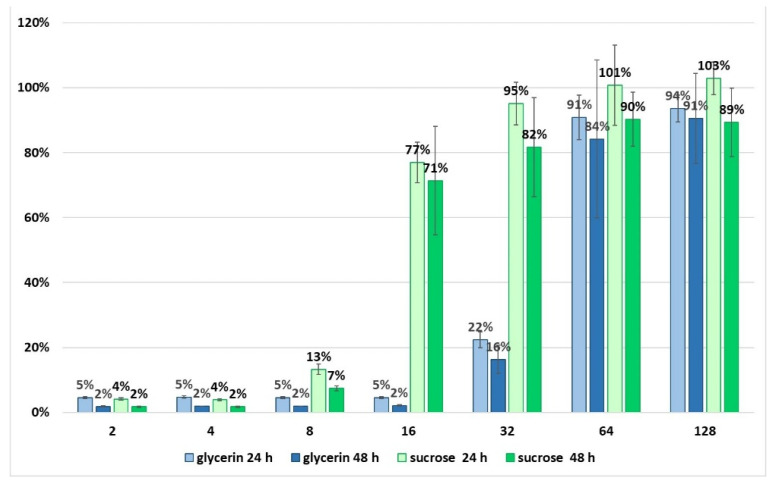
Viability of SC-1 R780 fibroblasts in the presence of glycerol or sucrose. The vertical axis denotes the percentage of viable cells, and the horizontal axis shows fold dilution of stock solutions of glycerol (75%) and sucrose (58%).

It turned out that when a 75% solution of glycerol is diluted 32-fold (down to 2.25% concentration)—Which corresponds to its calculated concentration when the final MLNC suspension is added to the cell culture—A negative effect on cell viability is detectable. By contrast, sucrose solutions did not manifest pronounced toxicity toward the cultured fibroblasts at the corresponding dilutions (Figure 11).

These findings indicate that the presence of glycerol in the culture medium, even at low concentrations, has an adverse effect on cells. Therefore, the MLNC preparations obtained using the purification method involving fractionation of samples in glycerol is not suitable for research on cultured cells. We present the results of this study to draw the readers’ attention to the necessity of comprehensive characterization before the approval of methods intended for nanobiotechnology and nanomedicine.

## 4. Conclusions

Lipid-coated particles that serve as a carrier of siRNA are the subject of numerous studies. Several years ago, we published the proof of principle for the construction of an AuNP-based MLNC that efficiently delivers siRNA into the cell [15]. Nonetheless, we were not satisfied with the quality of the obtained nanocomposites, and thus, here we found some ways to improve it in comparison with the original version. In this work, we demonstrated that even seemingly insignificant modifications of the steps of the MLNC fabrication affect end product quality. For example, the optimal reaction mixture for obtaining the core nanoparticles contains 0.1 mM MgSO_4_ and 5 mM NaCl; lipid film synthesis at 12 mmHg without thermostatting improves the quality of the forming MLNCs, as does the assembly of MLNCs in 1 mM phosphate buffer. Having optimized all the steps of MLNC fabrication, we noticed that 15 min centrifugation at 2000× *g* in 58% sucrose yields a fraction containing 40% of target MLNCs, i.e., a doubled proportion of these nanoconstructs as compared to the end product of the original procedure [15].

We think that this study can help researchers who design nanoconstructs based on metal nanoparticles coated with a lipid envelope.

## Figures and Tables

**Table 1:** Physicochemical parameters of MLNCs at different concentrations of phosphate buffer (PB).

MLNC Sample ID	PB Concentration, mM	PDI	Hydrodynamic Diameter, nm
1	100	0.203 ± 0.012	749.2 ± 177
2	10	0.256 ± 0.010	169 ± 88
3	3	0.275 ± 0.015	137.7 ± 65
4	1	0.209 ± 0.009	158.7 ± 74

**Table 2:** Physicochemical characteristics of the core nanoparticles in phosphate buffer containing different magnesium salts.

Sample	Magnesium Salt	ζ Potential, mV	Hydrodynamic Diameter, nm	PDI
Core nanoparticles	none	−44 ± 1	25 ± 9	0.212 ± 0.011
Core nanoparticles	0.1 mM Mg(Ac)_2_	−45 ± 1	27 ± 11	0.416 ± 0.041
Core nanoparticles	0.1 mM MgSO_4_	−39 ± 1	25 ± 9	0.266 ± 0.050
Core nanoparticles	0.4 mM MgSO_4_	−40 ± 2	90 ± 85	0.565 ± 0.316

**Table 3:** Physicochemical characteristics of optimized MLNCs after fractionation at 2000× *g* for 30 min.

Sample	Hydrodynamic Diameter, nm	PDI	Gold Content, %
Optimized MLNCs	152 ± 75	0.205 ± 0.008	-
Upper fraction	215 ± 108	0.198 ± 0.011	12
Middle fraction	397 ± 163	0.160 ± 0.005	32
Bottom fraction	1259 ± 461	0.097 ± 0.009	24
Total gold content			68

## Data Availability

Data are available on request from the corresponding author.

## References

[1] Kulkarni J.A., Witzigmann D., Thomson S.B., Chen S., Leavitt B.R., Cullis P.R., van der Meel R. (2021). The current landscape of nucleic acid therapeutics. Nat. Nanotechnol..

[2] Margus H., Arukuusk P., Langel Ü., Pooga M. (2015). Characteristics of Cell-Penetrating Peptide/Nucleic Acid Nanoparticles. Mol. Pharm..

[3] Elsabahy M., Nazarali A., Foldvari M. (2011). Non-Viral Nucleic Acid Delivery: Key Challenges and Future Directions. Curr. Drug Deliv..

[4] Zhou S., Chen W., Cole J., Zhu G. (2020). Delivery of nucleic acid therapeutics for cancer immunotherapy. Med. Drug Dicovery.

[5] Gupta A., Andresen A.J., Manan R.S., Langer R. (2021). Nucleic Acid Delivery for Therapeutic Applications. Adv. Drug Deliv. Rev..

[6] Berger M., Lechanteur A., Evrard B., Piel G. (2021). Innovative lipoplexes formulations with enhanced siRNA efficacy for cancer treatment: Where are we now?. Int. J. Pharm..

[7] Rinoldi C., Zargarian S.S., Nakielski P., Li X., Liguori A., Petronella F., Presutti D., Wang Q., Costantini M., De Sio L. (2021). Nanotechnology-Assisted RNA Delivery: From Nucleic Acid Therapeutics to COVID-19 Vaccines. Small Methods.

[8] Aghamiri S.H., Raee P., Talaei S., Mohammadi-Yeganeh S., Bayat S.H., Rezaee D., Ghavidel A.A., Teymouri A., Roshanzamiri S., Farhadi S.H. (2021). Nonviral siRNA delivery systems for pancreatic cancer therapy. Biotechnol. Bioeng..

[9] Shaabani E., Sharifiaghdam M., De Keersmaecker H., De Rycke R., De Smedt S., Faridi-Majidi R., Braeckmans K., Fraire J.C. (2021). Layer by Layer Assembled Cihitosan-Coated Gold Nanoparticles for Enhanced siRNA Delivery and Silencing. Int. J. Mol. Sci..

[10] Sharifiaghdam M., Shaabani E., Sharifiaghdam Z., De Keersmaecker H., De Rycke R., De Smedt S., Faridi-Majidi R., Braeckmans K., Fraire J.C. (2021). Enhanced siRNA Delivery and Selective Apoptosis Induction in H1299 Cancer Cells by Layer-by-Layer-Assembled Se Nanocomplexes: Toward More Efficient Cancer Therapy. Front. Mol. Biosci..

[11] Bonoiu A.C., Mahajan S.D., Ding H., Roy I., Yong K.-T., Kumar R., Hu R., Bergey E.J., Schwartz S.A., Prasad P.N. (2009). Nanotechnology approach for drug addiction therapy: Gene silencing using delivery of gold nanorodsiRNA nanoplex in dopaminergic neurons. Proc. Natl. Acad. Sci. USA.

[12] Bai X., Wang Y., Song Z., Feng Y., Chen Y., Zhang D., Feng L. (2020). The Basic Properties of Gold Nanoparticles and their Applications in Tumor Diagnosis and Treatment. Int. J. Mol. Sci..

[13] Li W., Cao Z.H., Liu R., Liu L., Lia H., Li X., Chen Y., Lu C.H., Liu Y. (2019). AuNPs as an important inorganic nanoparticle applied in drug carrier systems. Artif. Cells Nanomed. Biotechnol..

[14] Lopes T.S., Alves G.G., Pereira M.R., Granjeiro J.M., Leite P.E.C. (2019). Advances and potential application of gold nanoparticles in Nanomedicine. J. Cell Biochem..

[15] Poletaeva J., Dovydenko I., Epanchintseva A., Korchagina K., Pyshnyi D., Apartsin E., Ryabchikova E., Pyshnaya I. (2018). Non-Covalent Associates of siRNAs and AuNPs Enveloped with Lipid Layer and Doped with Amphiphilic Peptide for Efficient siRNA Delivery. Int. J. Mol. Sci..

[16] Tschuch C., Schulz A., Pscherer A., Werft W., Benner A., Hotz-Wagenblatt A., Barrionuevo L.S., Lichter P., Mertens D. (2008). Off-target effects of siRNA specific for GFP. BMC Mol. Biol..

[17] Pavlova A.S., Yakovleva K.I., Epanchitseva A.V., Kupryushkin M.S., Pyshnaya I.A., Pyshnyi D.V., Ryabchikova E.I., Dovydenko I.S. (2021). An Influence of Modification with Phosphoryl Guanidine Combined with a 20-O-Methyl or 20-Fluoro Group on the Small-Interfering-RNA Effect. Int. J. Mol. Sci..

[18] Shashkova V.V., Epanchintseva A.V., Vorobjev P.E., Razum K.V., Ryabchikova E.I., Pyshnyi D.V., Pyshnaya I.A. (2017). Multilayer Associates Based on Oligonucleotides and Gold Nanoparticles. Rus. J. Bioorg. Chem..

[19] Epanchintseva A., Vorobjev P., Pyshnyi D., Pyshnaya I. (2018). Fast and Strong Adsorption of Native Oligonucleotides on Citrate-Coated Gold Nanoparticles. Langmuir.

[20] Liu X., Atwater M., Wang J., Huo Q. (2007). Extinction coefficient of gold nanoparticles with different sizes and different capping ligands. Colloids Surf. B.

[21] Rodrı´guez-Corrales J.A., Josan J.A. (2017). Resazurin Live Cell Assay: Setup and Fine-Tuning for Reliable Cytotoxicity Results. Methods Mol. Biol..

[22] Epanchintseva A.V., Poletaeva J.E., Pyshnyi D.V., Ryabchikova E.I., Pyshnaya I.A. (2019). Long-term stability and scale-up of noncovalently bound gold nanoparticle-siRNA suspensions. Beilstein J. Nanotechnol..

[23] Epanchintseva A., Dolodoev A., Grigoreva A., Chelobanov B., Pyshnyi D., Ryabchikova E., Pyshnaya I. (2018). Non-covalent binding of nucleic acids with gold nanoparticles provides their stability and effective desorption in environment mimicking biological media. Nanotechnology.

[24] Menhaj A.B., Smith B.D., Liu J. (2012). Exploring the thermal stability of DNA-linked gold nanoparticles in ionic liquids and molecular solvents. Chem. Sci..

[25] Zhang Z., Li H., Zhang F., Wu Y., Guo Z., Zhou L., Li J. (2014). Investigation of halide-induced aggregation of Au nanoparticles into spongelike gold. Langmuir.

[26] Liu B., Kelly E.Y., Liu J. (2014). Cation-size-dependent DNA adsorption kinetics and packing density on gold nanoparticles: An opposite trend. Langmuir.

[27] Federal Medical & Biological Agency Research Institute of Physical-Chemical Medicine. Website of Extracellular Vesicles Research Group. Centrifugation Calculator. http://vesicles.niifhm.ru.

